# Clinical Manifestations of β-Thalassemia Major in Two Different Altitudes; Bushehr and Shahrekord 

**Published:** 2016-04-01

**Authors:** Mohammad Reza Ravanbod, Ali Movahed, Afshin Ostovar, Ali Hajigholami, Gholamreza Khamisipour, Shokrollah Farrokhi, Hossein Darabi, Yasaman Khosravi, Mohammad Kazzem Gheybi

**Affiliations:** 1 Assistant Professor, Department of Hematology and Oncology, Bushehr University of Medical Sciences, Bushehr, Iran; 2 Associate Professor, Department of Biochemistry, Bushehr University of Medical Sciences, Bushehr, Iran; 3 Assistant Professor, Department of Epidemiology, Persian Gulf Tropical Medicine Research Center, Bushehr University of Medical Sciences, Bushehr, Iran; 4 Assistant Professor, Department of Hematology and Oncology, Shahrekord University of Medical Sciences, Shahrekord, Iran; 5 Assistant Professor, Department of Laboratory Sciences, Bushehr University of Medical Sciences, Bushehr, Iran; 6 Department of Immunology, Persian Gulf Tropical Medicine Research Center, Bushehr University of Medical Sciences, Bushehr, Iran; 7 Persian Gulf Tropical Medicine Research Center, Bushehr University of Medical Sciences, Bushehr, Iran

**Keywords:** β-thalassemia major, Ferritin level, Cardiac function, Altitude

## Abstract

**Background:** Patients with β-thalassemia major (TM) develop iron overload through increased iron absorption and transfusional therapy and it’s the most important complication of TM. Thalassemia is common in coastal regions and lands with low altitudes. The aim of this study is to determine the effect of high and low altitude on serum ferritin and treatment requirement in two groups of β-thalassemia major (TM) patients.

**Subjects and Methods:** Patients were divided into two groups, the first group (No: 50) living at sea level (in the port of Bushehr, Iran) and the second group (No: 40) living at the altitude of 2061 m (in the city of Shahrekord, Iran). All patient’s clinical history, blood transfusion and laboratory tests including complete blood count and hemoglobin electrophoresis were reviewed.

**Results:** There were no significant difference in ferritin levels, transfusion period and diabetes incidence of the two cities patients (P>0.05). Patient’s cardiac function and liver condition were significantly better in patients of Bushehr (P<0.05). Patients under 20 years in Bushehr were less splenectomized in comparison with Shahrekord (P<0.05).

**Conclusion:** Our result showed that some of clinical manifestations of patients in low altitude such as cardiac and liver condition were better. But it did not affect ferritin level probably due to transfusion and chelating therapy. Totally patients of Bushehr had better conditions and had longer survivals.

## Introduction

 β-thalassemia major is an inherited form of hemolytic anemia , characterized by abnormal production of red blood cells. It is a worldwide hereditary blood disorder, highly prevalent in Mediterranean countries, Middle East, Central Asia, India, Southern China and the Far East as well as countries along the north coast of Africa and South America.^1^^,^^2^ It has been estimated that % 1.5 of world population are carriers of β-Thalassemia.^3^

The manifestations of the disease include myocarditis and iron overload which seem to be involved in the pathogenesis of left and right ventricular dysfunction and enlargement, respectively. Also, the mechanical efficiency of the heart in TM patients has been reported to decrease by arterial stiffness, endothelial dysfunction and LV hypertrophies.^4^ The manifestations of the disease are reported to be aggravated by hypoxemia. Erythropoietin in the blood circulation is sensitive to arterial blood O2 pressure and increases under hypoxic conditions.^5^ Therefore, at high altitudes where the oxygen pressure is reduced, the erythropoietin level in the blood rises and the total erythrocyte mass would increase which results in the early destruction of spleen and increase in the risk of splenectomy.

Transfusion therapy may increase iron absorption and results in iron overload in patients with TM, which is associated with endocrinopathies: hypogonadism, hypothyroidism, hypoparathyroidism, diabetes, liver fibrosis and heart dysfunction. Therefore, complicated and major focus in therapeutic management is iron overload.^6^^-^^8^ In addition, one of the causes of iron overload in TM patients is ineffective erythropoiesis,^9^^,^^10^ which leads to deficiency of vitamins and minerals.^11^^,^^12^

Thalassemia appears to be common in coastal regions and lands with low altitudes.^13^^,^^14^ However, Haldane in 1949 suggested that, the high frequency of thalassemia gene in the places surrounding the Mediterranean Sea might be a consequence of positive selection by endemic malaria.^15^^,^^16^ Although some studies demonstrated that in addition to malaria, other factors play a selective role in geographic disturbance of thalassemia.^17^ Moreover, the reports from one study has shown that protective role of β-thalassemia against G6PD deficiency is attributed to the oxidative plants grown in low land areas, which provoke fauvism in patients with TM. ^18^ The results from some studies have shown that β-thalassemia patients seemed to be highly sensitive to hypoxia at high altitudes.^16^^,^^18^ Hepcidin is an iron-regulatory hormone produced by hepatocytes which opposes iron absorption and erythropoiesis.^19^^,^^20^ Conditions such as iron overload, inflammation, anemia and hypoxia seem to decrease the production of this hormone which inhibits the effect of iron absorption and release of iron from macrophages, leading to increase in erythropoiesis.^21^ Another study has reported that Hepcidin might be reduced in healthy volunteers exposed to high altitude in both chronic and acute conditions.^22^ Moreover; it has been shown that low levels of Hepcidin in TM patients might be due to ineffective erythropoiesis activity^23^^,^^24^ which may be aggravate in high altitude.

The aim of this study was to compare the effects of hypoxia on clinical features of TM patients at high and low altitudes, with respect to heart and liver functions.

## SUBJECTS AND METHODS

 This is a multicenter, cross-sectional study conducted in Bushehr and Shahrekord cities in Iran on two groups of patients with β-thalassemia major. The first group was consisted of 50 patients and the second group was 40. They were matched by their sex and BMI. The first group was living in Bushehr Port in northern coast of Persian Gulf, at the sea level with barometric pressure of 1 atmosphere and oxygen pressure of 160 mmHg. Bushehr is one the most prevalent cities of Iran for β-thalassemia and is appropriate for this research. The second group was living in Shahrekord located at the highest altitude (2061 m from sea level) in central area of Iran, with barometric pressure of 600 mmHg, and oxygen pressure of 125 mmHg.

**Patients**

The patients were diagnosed by the clinical history, requirement for regular blood transfusion and laboratory tests including Complete Blood Count and Hemoglobin Electrophoresis. All selected patients were resident of the mentioned cities for at least 5 last years. The exclusion criteria were patients younger than 14 years old and those who had bone marrow transplant.

The study was approved by ethics committee of Bushehr University of medical sciences and written informed consent was obtained from all patients.

**Assessment of the clinical features**

A stadiometer was used to measure height and weight. Body mass index was calculated. A questionnaire consisted of the characteristics and clinical features of the patients were completed by the help of an expert in the field. Information like transfusion requirement, splenectomy and diabetes history, puberty symptoms like menstruation or ejaculation, bone fracture history and sleeping quality were checked from patients file in thalassemia ward or asked verbally and recorded.

The average blood ferritin level of the TM patients measured during the previous one year (measured every two months by ELISA method) was obtained from thalassemia ward records. The patient’s cardiac function and hepatic status were evaluated by using echocardiography and magnetic resonance imaging respectively. General health questionnaire-28 was also used for assessing patient’s General psychological health.

**Statistical analysis**

Probability values <0.05 were considered statistically significant. The significance of the difference in the results between the two groups was determined with chi-square analysis using 2*2 contingency tables. A two-tailed t-test was used to compare the values across groups in the presence of a normal distribution. Spearman correlation analysis was employed to study the relationships among serum ferritin levels and patients age. All statistical analysis was performed using the PASW Gradpack 21 (SPSS Inc. Chicago, IL).

## Results

 The study consisted of 50 patients from Bushehr (26 male, 24 female) and 40 patients from Shahrekord (22 male, 18 female). The sex and age were matched and there was no difference in the two groups (p=0.777). The mean ferritin level in patients of Bushehr and Shahrekord was 1395.90 ng/mL and 1206.59 ng/mL, respectively (Table 1). There was no significant difference in ferritin levels of two groups (p=0.296). There was no correlation between age and ferritin level (β=0.16, p=0.129).

**Figure 1:**
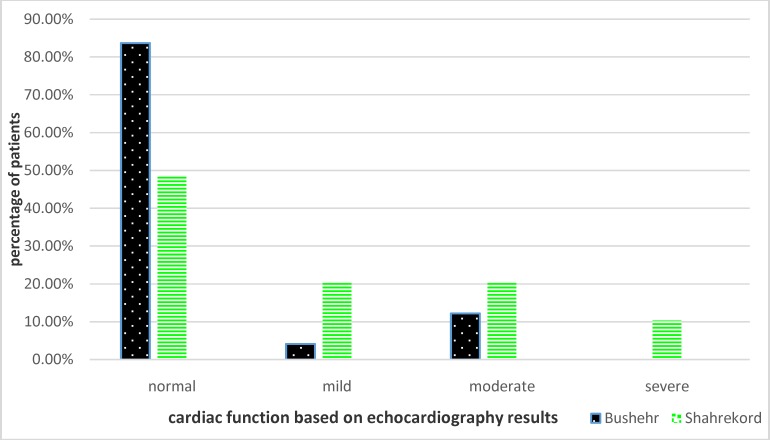
Cardiac function in Thalassemia major patients of Bushehr and Shahrekord based on the echocardiography results

The mean transfusion period for patients in the first and second group was 20.74 and 23.08 days, respectively. There was no difference in their transfusion period between the two groups (p=0.072). It was found that cardiac function was significantly better in patients living in low altitude place (p=0.001). Also, after correction for age and comparing patients in age groups, a significant difference was noticed in cardiac function of the patients under the age of 20 (p=0.034) (Figure 1).

**Figure 2:**
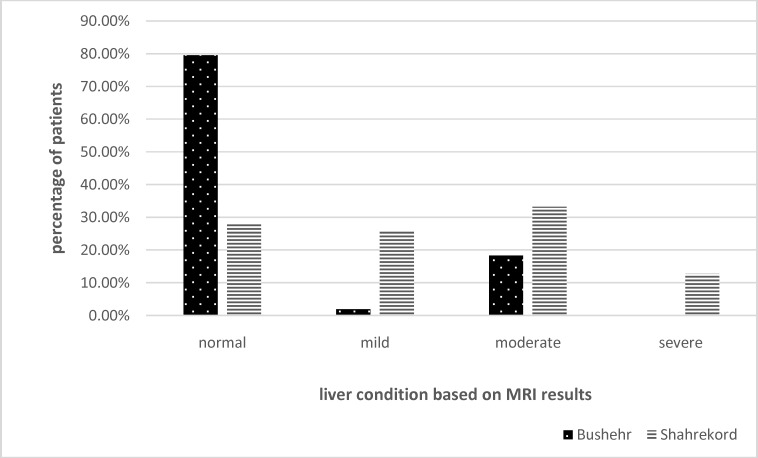
Liver condition in Thalassemia major patients of Bushehr and Shahrekord based on MRI of liver

We found that hepatic status was significantly better in patients of the first group (p=0.000) and after correction for age, there was a significant difference in liver function of the patients under the age of 24 years living in low altitude city (p=0.008) (Figure 2). There was no difference between incidence of diabetes in both groups (p=0.129). However, 11 patients from Bushehr city and 4 patients of Shahrekord suffered from diabetes. The result of this study showed that 20 patients from group 1 and 22 from group 2 were splenectomized. However, no significant difference was observed between the two groups (p=0.156). After correction for age and comparing patients in age groups, it was found that the patients under the age of 20 from the first group were less splenectomized (1 out of 20), than the patients of the same age in the second group (8 out of 14). During the study 2 patients living in high altitude died.

## Discussion

 The purpose of this study was to compare the clinical features of TM patients in two different altitudes and find out whether low altitude has any positive effect on the disease process. Regarding the fact that Bushehr and Shahrekord are both located in Iran and the protocols that physicians use for their therapy and observation are the same. The results of this study showed no significant difference in serum ferritin level between the two groups.

**Table 1:** Demographic and Clinical information of patients with β-thalassemia major

	**Bushehr ** **(n=50)**	**Shahrekord ** **(n=40)**	**P value**
**Sex**	M=26 F=24	M=22 F=18	0.777
**Mean Age (yrs)**	23.8 (14-41)	21.33 (14-29)	0.156
**Mean Ferritin (ng/ml)**	1395.9	1206.59	0.296
**Intervals of Transfusion (days)**	20.74	23.08	0.072
**Number 0f Diabetes**	11	4	0.129
**Total cases of splenectomy**	20	22	0.156
**Splenectomy cases under age 20yrs**	1 of 20	8 of 14	*0.007

**F:** (female), **M:** (male), **yrs** (years), **ng/ml: **(nanogram per mililitre), *: significant

There were also no higher ferritin levels in older patients although they had transfusional therapy for a longer time. Since serum ferritin has been used to estimate iron overload,^9^ it was expected that the patients living in low altitude to have lower ferritin levels. The cause is, iron overload in these patients is not only depended to ineffective erythropoiesis and iron absorption but is mostly influenced by their transfusion diet and chelating therapy.

Transfusion interval times in TM patients living in Bushehr were less than those in Shahrekord however, the difference was not significant. In this study, the transfusion diets were based on patient’s job and request. Other factors such as BMI or hemoglobin level were given low priority or ignored. Previous studies have shown that cardiac complications may be the cause of 50-70 percent of death in TM patients.^25^ Cardiac output measurement has been recommended for the assessment of cardiac function.^26^ Therefore, left ventricular ejection fraction (EF) above % 60 considered to be normal.^27^ The results of the present study showed that, many of the patients living in low altitude had EF of above 60%. After adjusting age, better cardiac condition was also seen in younger patients living in low altitude. However, no difference was noticed in older patients. It may probably be due to long time transfusion and chronic cardiac iron overload.

Diabetes and insulin resistance are common issue in TM patients (6-10%).^28^ We observed 16.6% prevalence of diabetes in our study but low altitude of living place had no relation with incidence of diabetes.

MRI examination of the liver used to assess hepatic fibrosis and has been regarded as the golden standard for the total body iron overload.^26^ In the present study patients living in lower altitude had better hepatic status and after adjustment for age, this was also true in younger patients. According to previous studies liver iron overload worsens cardiac disease in TM patients and this can be an explanation for the difference in cardiac condition of the two groups.^29^ Splenomegaly and hypersplenism have been shown to be common in TM; they occur due to the excessive storage of iron.^30^^,^^31^ In addition, splenectomy is a way to reduce excessive blood consumption.^32^ In this study, no difference was found in the occurrence of splenectomy in the two groups. However, after adjusting for age, it was shown that younger patients living in high altitude experienced higher occurrence of splenectomy as compared to those in low altitude. The cause might be due to lack of sufficient therapeutic drugs and blood transfusion in the past decades; so, the spleen used to be removed in older TM patients routinely. However, in the present time splenectomy may be required only under conditions like increased transfusion requirement and hypersplenism.^32^ In addition to following results none of the patients living in higher altitude had the age of more than 29 as 2 of them passed away during 18 month of our research period but there were plenty of patients more than 30 and 40 living in low altitude.

The patients with TM living in high altitude probably experience lower levels of hepcidin in their blood circulation, leading to ineffective erythropoiesis which causes iron overload and life threatening dysfunctions. Although the low hepcidin level has been reported to rise after transfusion, it starts to fall again later.^24^ Since there was no transfusion or chelating therapy in the past centuries, TM patients probably developed ineffective erythropoiesis in very higher levels especially in higher lands and those patients would not have survived. So altitude might have played a selective role in geographic disturbance of thalassemia its self.

One of the limitations of this study was the small size of the population under study. So, more studies with greater population and more specific in clinical features seems to be required to define the role of altitude in disease process of TM. Also, direct measurement of hepcidin before and after transfusion is suggested.

## CONCLUSION

 The results of present study showed that living in low altitude may improve cardiac and liver function of TM patients. However there were no changes in ferritin level between the two groups which was suggested to be due to routine transfusion and chelating therapy.
